# Frozen shoulder: overview of clinical presentation and review of the current evidence base for management strategies

**DOI:** 10.2144/fsoa-2020-0145

**Published:** 2020-10-30

**Authors:** Akshay Date, Luthfur Rahman

**Affiliations:** 1 Department of Orthopaedics, Basildon & Thurrock University Hospital, Nethermayne, Basildon, Essex, UK, SS16 5NL, UK; 2 Department of Orthopaedics, Chelsea & Westminster Hospital, 369 Fulham Rd, Chelsea, London SW10 9NH, UK

**Keywords:** adhesive capsulitis, frozen shoulder, management, pathophysiology

## Abstract

Adhesive capsulitis of the shoulder (ACS) is a condition with significant clinical and economic implications. The etiology of adhesive capsulitis is not clearly understood and there remains lack of consensus in clinical management for this condition. It can occur as a primary idiopathic condition or secondary to medical conditions or trauma. The hallmarks of ACS are pain and stiffness, caused by formation of adhesive or scar tissue in the glenohumeral joint. Management strategies vary depending on stage of presentation, patient factors and clinician preferences, and can range from conservative options to surgical intervention. The aim of this review is to summarize the pathophysiology and clinical presentation of ACS and to discuss the evidence base for various management strategies employed today.

## Lay abstract

Frozen shoulder (FS) is a relatively common condition characterized by pain and stiffness of the shoulder joint. The exact cause of primary FS is unknown and in some patients the condition can persist for several years. Treatment strategies vary depending on stage of presentation, patient factors and clinician preferences. This review gives a summary of the clinical presentation of FS and an overview of the current evidence for both surgical and conservative treatment options for the condition.

Adhesive capsulitis of the shoulder (ACS) was first described by Duplay [1] in 1872 as ‘periarthritis’ and subsequently by Codman [2], who coined the term ‘frozen shoulder’ in 1934 [3]. ACS is characterized by varying degrees of pain and restricted movements of the glenohumeral joint. It occurs in approximately 2–5% of the population with a peak incidence between 40 and 70 years of age. It is more common in females and bilateral in 20–30% of cases. The condition is usually self-limiting; however, in some patients, symptoms can last for several years or may never fully resolve [4]. Treatment for ACS remains a challenge today, placing a significant burden on healthcare systems as well as the wider economy, with patients unable to work for prolonged periods of time. This review article aims to give a brief overview of the pathophysiology and clinical presentation of ACS and to summarize the evidence base for conservative and surgical management of the condition [5].

## Pathophysiology & clinical presentation

Adhesive capsulitis can be either primary or secondary; secondary causes of ACS include trauma, previous shoulder surgery, prolonged immobilization [6], diabetes [7], thyroid disease [8], Dupuytren disease [9] and other autoimmune disorders [10]. Primary idiopathic frozen shoulder occurs in patients presenting with painful, restricted shoulder movements where no underlying cause is found. The pathophysiology of primary adhesive capsulitis is still not fully understood, but histological studies have shown that frozen shoulder is characterized by a thickened, tight capsule, with chronic inflammatory cells and fibroblasts found in the joint capsule [11] Furthermore, fibroblasts in frozen shoulder have an activated phenotype associated with cytokine dysregulation, suggesting an autoimmune etiology [12,13].

Clinically, four stages of adhesive capsulitis have been described as a useful method of monitoring and assessing symptomatology [14] [Table 1]. The condition starts with a painful phase, characterized by pain and progressive restriction of movement. This phase is characterized by hypertrophic synovitis with hypervascularity, but a normal appearance of the capsular tissue [15]. This is followed by a ‘freezing’ stage where symptoms gradually worsen over 9 months; histologically, there is perivascular synovitis and collagen deposition. Over the subsequent 1–4 months, the ‘frozen’ phase occurs, characterized by stiffness as a predominating symptom. The ‘thawing’ phase, in which symptoms resolve, has the greatest variability in duration and can last for up to 2 years. The latter two stages involve the formation of dense collagenous tissue in the capsule, associated with scar formation [15].

**Table 1.** Clinical stages of ACS and histological findings.

Stage		Symptoms	Duration of symptoms (months)	Histology
I	Painful stage	Moderate pain and reduction of movement	<3	Hypertrophic synovitis with hypervascularity Normal capsular tissue
II	Freezing stage	Severe pain and reduction of movement	3–9	Perivascular synovitis and disorganized collagen deposition and scarring
III	Frozen stage	Pain may be present but stiffness predominates	10–14	Dense and hypercellular collagenous tissue of the capsule
IV	Thawing stage	Minimal pain and gradual improvement in movement	14–24	Fully developed scar tissue Pathophysiology remains unclear

There is significant variation in clinical practice and the disease course may not follow such a stepwise progression. The diagnosis is usually made clinically, with pain and stiffness as the hallmark of the condition. The onset of pain is often gradual over a period of months, with night pain being a common feature. The pain may be poorly localized and described as a deep ache, or sometimes presents as a pain referred to the deltoid origin, radiating to the biceps area. Examination findings are often nonspecific without any point tenderness and with normal rotator cuff strength. However, both passive and active range of motion (ROM) are globally reduced; this is best assessed through passive external rotation with the arm by the side. The results of laboratory tests are usually normal but may be useful in identifying underlying conditions, such as diabetes or thyroid disease. Plain radiographs of the shoulder are also usually normal but can help diagnose or exclude other conditions, such as calcific tendinopathy of the rotator cuff, glenohumeral arthritis, acromioclavicular arthritis or even a shoulder dislocation. In calcific tendinopathy, disuse osteopenia may be demonstrated on the plain radiographs. Imaging modalities such as arthrography, technetium bone scans [16] and magnetic resonance imaging [17] are not routinely indicated or helpful in evaluation of adhesive capsulitis but can be used to exclude other shoulder pathology [18].

## Management

The goal of treatment in ACS is to restore function and manage symptoms. The choice of treatment can vary with patient factors, stage at presentation, clinician preferences and local policies or funding. Nonsurgical or conservative management is the preferred choice of treatment, with most patients usually improving in 6–18 months [19]. Conservative treatment options include analgesics, oral steroids, physical therapies, hydrodilatation, suprascapular nerve block (SNB) and intra-articular steroid or sodium hyaluronate injections. Surgical treatment is offered to patients with persistent symptoms despite conservative management; strategies include manipulation under anesthesia (MUA), arthroscopic release and open release. There remains no consensus or high-level evidence to definitively support one treatment modality over another.

## Nonsurgical treatment

### Physical therapy

Physical therapy has traditionally been the initial treatment modality in ACS and it is often utilized alongside other adjuncts, including steroid injections, transcutaneous electrical nerve stimulation, analgesics and warm or cold pads [20,21]. There remains variation of physical therapy regimen in both clinical practice and in the literature; however, the principles revolve around a supervised stretching and strength maintenance program [22,23]. In the early freezing stage, gentle stretching exercises of short duration are recommended, including pendulum exercises and passive external supine forward elevation [24]. Strengthening exercises such as isometric shoulder external rotation and posterior capsular stretching can be introduced in the frozen stage [24]. In the thawing stages, both strengthening and stretching exercises can be combined and increased in frequency, or combined with Maitland Grade III–IV mobilization, to improve ROM. Griggs *et al.*, in a prospective nonrandomized study of 75 patients, showed that a supervised stretching program resulted in a satisfactory outcome in 90% of patients at 22 months [25]. Vermeulen *et al.* [26] showed that high-grade mobilization (working through the pain barrier) was marginally better than low-grade mobilization (working within pain limits).

Despite the clinically prevalent use of physical therapy in ACS, there remains a lack of high-level evidence to support physical therapy over observation or medical therapy alone [18,27]. Randomized controlled trials (RCTs) by Bulgen *et al.* [28] and later by Carette *et al.* [29] showed no significant difference between patients who had physical therapy and those receiving no treatment. Further research is required to determine the role for physical therapy and adjuncts to treatment in ACS management.

### Pharmacological treatments

Paracetamol and NSAIDs are often used as first-line treatments for pain in adhesive capsulitis. Analgesics may be more effective when combined with physical therapy [20] and although NSAIDs have been shown in some studies to provide significant improvement of symptoms compared with placebo [30], there is no high-level evidence to support this. Studies comparing different NSAIDs including naproxen and indomethicin have shown no significant differences in their effect [31,32].

Oral steroids have also been shown to have benefit in ACS; Buchbinder *et al.* [33] showed in a double-blind, placebo-controlled RCT that patients taking 30 mg oral prednisolone daily as a 3-week course had greater improvement in disability, range of active motion and overall pain compared with placebo groups (p = 0.001). However, there was no significant difference between the groups beyond 6 weeks; the authors recommended prednisolone for its short-term benefits. Similar findings were reported by Binder *et al.* [34], who showed in a study of 40 patients that night pain significantly improved in patients receiving oral prednisolone over the short term, but that there were no differences between groups at 8 months. There may be a role for oral steroids in ACS in providing short-term pain relief and ROM improvement; however, clinicians should exercise caution with the long-term use of steroids due to the high side effects profile and limited evidence in the literature to support their use in ACS for a prolonged period.

### Intra-articular steroid injections

Intra-articular steroid injections are routinely used in the management of ACS and have been shown to provide better short-term pain relief and improved movement compared with oral steroids [35,36] A recent meta-analysis with 225 patients showed that intra-articular steroid injections provided short-term analgesia (reduced pain score up to 8 weeks) and improved passive ROM in both short and long term (up to 24 weeks), compared with control groups receiving placebo [37]. Furthermore, a systematic review identified three high-quality RCTs showing a significant analgesic benefit of intra-articular injections, given between 6 weeks and 4 months, over placebo or physiotherapy alone [38]. Shah *et al.* reported that multiple injections are efficacious without causing significant complications [39]. Ultrasound-guided injections may be preferred because injections without radiological guidance have a high incidence of not entering the glenohumeral joint [40,41]. However, recent evidence also suggests there may be no significant difference in outcomes between subacromial and glenohumeral injection [42,43].

There is substantial high-level evidence in the literature comparing steroid injections with physiotherapy. Koh *et al.*, in a systematic review of RCTs, report corticosteroid injections as superior to placebo and physiotherapy in the short term (12 weeks) [43] Furthermore, a meta-analysis of nine RCTs with 453 patients found steroid injections equally as effective as physiotherapy; the authors recommend single steroid injection as the first line treatment in ACS [44]. However, several other RCTs have shown that steroid injections used as adjuncts to physiotherapy provide greater improvement in symptoms when compared with physical therapy alone [29,45,46].

### Intra-articular sodium hyaluronate injections

Sodium hyaluronate can be used as an alternative to steroid injections [47] or indeed combined with steroids for the treatment of adhesive capsulitis [48]. A systematic review of seven studies with 140 ACS patients treated with hyaluronate injections showed hyaluronate to be better than placebo and as effective as corticosteroid injections, with the added benefit of fewer side effects [49]. In addition, it has been suggested that sodium hyaluronate may have a chondroprotective effect and improve the properties of synovial fluid [50]. A randomized study of 30 patients found tramadol with hyaluronate to be more effective than hyaluronate injections alone [51]. However, there remains controversy in the literature, with Lee *et al.* showing in their review of four RCTs including 273 patients that intra-articular hyaluronate was not superior to corticosteroid injection or physical therapy [52].

### Hydrodilatation

Intra-articular distension of the shoulder joint is an alternative method of treatment which has been shown to be of benefit but predominantly in the short term and has not been shown to have any significant benefit in the long term over other treatment modalities. The technique involves dilation of the capsule with either saline or steroid and local anesthetic in an attempt to stretch the capsule and break down any adhesions [53]. Quraishi *et al.* [54], in a randomized trial of 36 patients, compared hydrodilatation with manipulation under anesthetic and demonstrated improved functional scores over a six-month period (p = 0.02) in the hydrodilatation group. There was improvement but not statistical significance in ROM at 6 months in both groups.

Three RCTs have compared hydrodilatation with intra-articular steroid injection. Gam *et al.* [55] reported a significant improvement in ROM in the hydrodilatation group but pain and functional scores were comparable; ROM is not a validated outcome for ACS and the findings from this study should be interpreted with caution. Similarly, Tveita *et al.* [56] and Corbeil *et al.* [57] found no difference in outcomes of pain and ROM. In addition, one study by Khan *et al.* [58] compared hydrodilatation with physical therapy; ROM for abduction and external rotation was significantly improved at 8 weeks with hydrodilatation and physiotherapy compared with physiotherapy alone. However, once again there was no significant improvement demonstrated in pain score. The studies described here were limited by sample size and randomization, and the heterogeneity of study design makes it difficult to draw confident conclusions [55–58]. There is insufficient evidence in the literature to suggest hydrodilatation as superior to other treatment modalities in ACS.

### Suprascapular nerve block

The supraclavicular nerve arises from the superior trunk of the brachial plexus (C5, C6) and supplies the muscles of the rotator cuff, namely the supraspinatus and the infraspinatus. SNB involves local anesthetic infiltration through a needle inserted behind the lateral end of the clavicle at the insertion of the trapezius [59]. The effectiveness of SNB may be improved using electromyographic or ultrasound guidance [60–62]. In a double-blind, placebo-controlled RCT of 34 patients, Dahan *et al.* [63] demonstrated a significantly greater improvement in pain (p = 0.03) but no difference in function (p = 0.24) after SNB with bupivacaine compared with placebo at 1 month. Similarly, Jones *et al.* performed a prospective RCT and showed that SNB may also provide quicker and more complete resolution of symptoms compared with intra-articular steroid injections [62]. Mortada *et al.* showed in their trial of 96 patients that a course of nine injections gave a better outcome than a single injection [64]. The literature suggests that SNB could be an effective short-term strategy in ACS, although larger multicenter trials would be useful in further defining its role.

## Surgical treatment

### Manipulation under anesthesia

Surgical treatments are usually reserved for patients who have persistent symptoms despite nonoperative treatment for between 2 and 6 months [65–67]. Manipulation of the shoulder is a technique in which the patient is anesthetized and the joint is manipulated to break down thickened and stiff capsular tissue. MUA has been shown to have good results in both the short and the long term, particularly when followed with a program of physical therapy [68,69]. MUA may also be combined with interscalene blocks, which have been shown to provide a sustained improvement in both function and movement at 12 months [70]. Furthermore, Farrell *et al.* demonstrated long-term improvements up to 15 years after MUA [71].

There are, however, significant complications to consider with MUA; these include humeral fractures, glenoid fractures, glenohumeral joint dislocation, rotator cuff tears (especially subscapularis), biceps tendon injuries and labral tears. Care needs to be taken when performing the procedure to avoid these complications while performing an effective manipulation to break down the inferior capsule. Manipulation techniques vary in clinical practice and in the literature, as described by Kraal *et al.* in their review [72].

### Arthroscopic release

Arthroscopic release is often the preferred method of surgical treatment in refractory cases of adhesive capsulitis. It was first described in 1979 by Conti [73] and helps avoid the potential but significant complications of MUA. Arthroscopic release has been shown to have good short- and long-term outcomes in terms of both pain and function [74,75]. Arthroscopic release is also effective in patients with insulin-dependent diabetes mellitus, although the outcomes are better in nondiabetic patients [76] Similarly, patients with idiopathic and posttraumatic adhesive capsulitis have better outcomes than patients with postoperative adhesive capsulitis [77].

There are several other advantages to arthroscopic release, including controlled release of contracted capsule, synovectomy (if necessary), direct visualization of the joint and identification of other shoulder pathology. In addition, postoperative physical therapy can be started early because patients often have minimal pain. There are, however, some  potential complications, including pain and recurrent stiffness, anterior dislocation of the shoulder and axillary nerve injury.

Although arthroscopic release has been shown to have good outcomes, there is still some controversy as to the surgical technique, particularly relating to the extent of the surgical release. Ogilvie-Harris *et al.* reported good pain relief and functional improvement after arthroscopic release of the intra-articular part of the subscapularis (IASS), superior glenohumeral ligament and the rotator interval [74]. Pearsall *et al.* reported that the IASS can be released during arthroscopic capsular release for frozen shoulder with minimal risk of secondary anterior instability [78]. However, the need to release the IASS is debatable and there is no definitive evidence to demonstrate that it confers significantly better outcomes. Snow *et al.* demonstrated no significant difference in improvement of ROM with both anterior and posterior capsular release compared with anterior release alone [79]. Conversely, Jerosch suggested that a 360-degree circumferential capsular release was a reliable method for restoring motion with minimum morbidity [80].

Kanbe *et al.* divided 267 ACS patients who underwent arthroscopic release into three groups, based on the severity of the adhesion between the coracohumeral ligament and the long head of biceps (LHB) tendon [81]. At 5 years post-surgery, all groups had significantly improved functional scores; however, patients with severe LHB and coracohumeral adhesion had significantly different outcomes from those with less severe adhesions (p < 0.0001) [81]. Notably, the authors also found diabetes mellitus to be a significant risk factor for severity of adhesion. This study highlights the importance of intraoperative release of LHB in arthroscopic release surgery for ACS.

### Open release

Open release for adhesive capsulitis is an uncommon procedure because arthroscopic surgery carries significantly less postoperative morbidity. It may, however, be an option in patients who have had unsuccessful arthroscopic release; it has been shown to produce good outcomes in terms of both pain and ROM [82,83], although diabetic patients had poorer outcomes [82]. Omari *et al.* showed functional and symptomatic improvement after open arthroscopic release in 25 patients who had failed MUA [84]. Additional indications for open release include patients who have suffered strokes or head injuries and in posttraumatic or postsurgical adhesive capsulitis where there are significant adhesions and contractures preventing arthroscopic surgery [85].

## Conclusion

ACS is a common condition that causes significant and prolonged morbidity for patients and carries wider economic implications. The management of adhesive capsulitis remains a challenge and there is a need for high-level, definitive evidence to suggest one form of treatment over another. As such, the treatment of this condition remains varied in clinical practice. There is moderate evidence for conservative approaches in the initial stages of ACS, including analgesics, oral steroids, intra-articular steroid or sodium hyaluronate injections, SNB and physiotherapy. Evidence in the literature for the efficacy of hydrodilatation therapy remains inconclusive. In cases resistant to conservative management strategies, MUA or arthroscopic capsular release are evidence-based treatment alternatives. There is limited evidence for open release, and it is also associated with greater post-operative morbidity compared with arthroscopic release. Surgical treatments should be complemented with an appropriate, supervised physical therapy regimen. Further understanding of the underlying pathology of ACS and large, randomized multicenter studies are required in order to define an evidence-based management strategy for the condition.

## Future perspective

The last two decades have seen significant progress in our understanding of the pathophysiology behind ACS, which has complemented developments in current management strategies. However, there remains variation in clinical practice with no consensus in treatment for this condition. Nonoperative interventions remain first-line approaches in managing ACS and there is an urgent need for prospective RCTs comparing the efficacy of different treatment strategies. Furthermore, as our understanding of the immunological etiology of ACS develops, there may be opportunities for immunomodulating or targeted therapies in the future.

**Executive summary**

- Adhesive capsulitis of the shoulder (ACS) is a relatively common condition characterized by pain and stiffness of the shoulder joint.
- The pathophysiology of ACS is still not fully understood; however, histological studies have shown that the condition is characterized by a thickened, tight capsule, with chronic inflammatory cells and fibroblasts found in the joint capsule.
- Clinically, the presentation can be classified into four stages: painful, freezing, frozen and thawing.
- The goal of treatment is to restore patient function and manage symptoms, with a myriad of nonoperative and operative treatment strategies used in clinical practice.
- Physical therapy, often with adjuncts, is the preferred first-line treatment, although there remains a lack of high-level evidence in the literature to support this approach.
- Oral steroids have been shown to be effective in the short term, although their benefits after 6 weeks remain doubtful.
- Steroid injections have been shown in the literature to be as effective as, or even superior to, physiotherapy in the management of ACS.
- Sodium hyaluronate has been shown in one systematic review to be as effective as steroids and more effective than placebo; however, the evidence in the literature is controversial.
- There is a need for further research on the effectiveness of suprascapular nerve block and hydrodilatation, with some studies demonstrating promising results.
- Surgical treatment strategies of manipulation under anesthesia and arthroscopic release are effective in persistent cases of ACS, although there is variation in technique in both clinical practice and literature.
